# Dynamical intervention planning against COVID-19-like epidemics

**DOI:** 10.1371/journal.pone.0269830

**Published:** 2022-06-14

**Authors:** Gabriele Oliva, Martin Schlueter, Masaharu Munetomo, Antonio Scala

**Affiliations:** 1 Unit of Automatic Control, Department of Engineering, Università Campus Bio-Medico di Roma, Rome, Italy; 2 Information Initiative Center, Hokkaido University, Sapporo, Japan; 3 CNR-ISC, Applico Lab, Roma, Italy; 4 Centro Ricerche Enrico Fermi, Roma, Italy; 5 Big Data in Health Society, Roma, Italy; 6 Global Health Security Agenda - GHSA, Roma, Italy; Frankfurt Institute for Advanced Studies, GERMANY

## Abstract

COVID-19 has got us to face a new situation where, for the lack of ready-to-use vaccines, it is necessary to support vaccination with complex non-pharmaceutical strategies. In this paper, we provide a novel Mixed Integer Nonlinear Programming formulation for fine-grained optimal intervention planning (i.e., at the level of the single day) against newborn epidemics like COVID-19, where a modified SIR model accounting for heterogeneous population classes, social distancing and several types of vaccines (each with its efficacy and delayed effects), allows us to plan an optimal mixed strategy (both pharmaceutical and non-pharmaceutical) that takes into account both the vaccine availability in limited batches at selected time instants and the need for second doses while keeping hospitalizations and intensive care occupancy below a threshold and requiring that new infections die out at the end of the planning horizon. In order to show the effectiveness of the proposed formulation, we analyze a case study for Italy with realistic parameters.

## Introduction

The ongoing COVID-19 pandemics, with its huge toll in terms of deaths and economic damage, represents an unparalleled global threat to human society as a whole. As of May 2021, reportedly more than 155 million COVID-19 cases have been identified, with more than three million deaths [1]. To face such a threat, governments have initially reacted via non-pharmaceutical interventions, i.e., by enforcing strict social distancing [2–5]. Then, with an unprecedented effort by human society as a whole, a wide variety of vaccines have been developed in a remarkably narrow time span [6–10]; such a rapid development has been possible also due to the extensive reliance on bioinformatics [7] and artificial intelligence [11]. Notably, the effectiveness and geographical distribution of such a plethora of vaccines has proven to be highly heterogeneous (e.g., see [12] and references therein). Notice that, to date, no universally acknowledged cure has been identified; the case of the debate regarding therapies based on Remdesivir represents an illustrative example in this sense [13, 14]. Therefore, to date, the control knobs available to governments amount to just social distancing (e.g., lockdowns, limiting affluence to shops, wearing masks, etc.) and vaccination. In the literature, optimization tools to face epidemics, such as OpenMalaria [15, 16] and STDSIM [17], have proved their effectiveness in analyzing and planning illness containment [18–20].

However, in the case of COVID-19, the unavailability of reliable information with adequate level of detail requires reliance on simpler approaches. Among several other options, SIR models [21, 22] represent a reasonable choice in terms of predictive capability and simplicity. Interestingly, such models have been applied to describe different epidemics such as SARS [23] Influenza A (H1N1) [24], Measles [25] and Hepatitis C [26]. Moreover, compartmental models in general have proven useful to model quite different epidemics scenarios e.g., Chlamydia trachomatis, antiviral treatment in the case of HIV, nosocomial infections and transmission of antibiotic-resistant pathogens [27]. For this reason, such model allows to understand the available degrees of freedom, i.e., the policies that can be put in place to react to the epidemics, even in the absence of detailed quantitative predictions [28]. Indeed, several control [29–31] and optimization [29, 32–35] approaches have been developed, based on simple epidemics models such as the SIR. In particular, it is worth mentioning: [35], where a coarse-grained and static optimization framework for selecting the amount of vaccines to be allocated to different population classes with the aim of ending the epidemics is given; [36], where the authors focus on vaccination of essential workers; [37] where the authors allocate vaccines to age classes in order to optimize cost functionals such as deaths or hospitalization, but do not guarantee the end of the epidemics at the end of the considered time horizon nor consider different vaccines with different efficacies at the same time. Other examples include [38, 39], where optimization of the supply chain underlying the vaccine delivery is considered. Notably, based on epidemiological data from the UK together with estimates of vaccine efficacy, [40] provides a framework to conduct “what if” analysis of the evolution of the disease under different interventions. However, while being beneficial for high-level policy making, does not translate into an operative plan for the administration of pharmaceutical and non-pharmaceutical interventions. Moreover, the workin [41] provides a model predictive approach to the problem where interventions at time t are selected based on the foreseen effect in the next future, following a “receding horizon” approach. However, although quite detailed, the epidemics model underlying such framework amounts to a single population of individuals and does not distinguish between age classes.

To the best of our knowledge, to date no formulation is able to support the fine-grain, operative and dynamical planning of interventions, while accounting for a wide range of phenomena that are peculiar of epidemics like COVID-19, e.g., different types of vaccines, the need for a second dose, the capacity of the healthcare system in terms of regular and intensive care hospitalization, the availability of vaccines in batches at selected time instants.

In this paper, we fill this gap by providing a novel optimization formulation that aims at implementing an optimal intervention plan to fight newborn epidemics like COVID-19, i.e., epidemics characterized by two key factors: (i) high infection rate and (ii) high stress posed on the healthcare system and/or society in terms of intensive care occupancy, deaths or economic consequences. Specifically, we first develop a modified discrete-time SIR model for heterogeneous population classes (e.g., age or geographical classes) that accounts for the effect of social distancing and vaccination. In more detail, we assume the ratio at which individuals in two classes infect each other can be reduced by enforcing tailored social distancing measures. Moreover, we consider several types of vaccines, each characterized by their efficacy as well as the delay required for the vaccination to be effective. In this view, we assume that, after the vaccine takes effect, a fraction of the population becomes immune. Notice that we explicitly take into account the possibility to plan for a first and a second dose of the vaccines.

Based on the proposed variation of the SIR model, we develop an optimization formulation that aims at planning social distancing measures and vaccinations at the level of the single day in order to reach the end of the epidemics and to guarantee that the state reached is robust, in that new infections die out. In doing so, we enforce constraints accounting for aspects such as the need of a second dose, the delayed and partial effect of multiple types of vaccines, the requirement that congestions of the healthcare system are avoided.

Briefly, we show that the proposed formulation amounts to a *Mixed Integer Nonlinear Programming* (MINLP) problem. We point out that the term MINLP is used to denote a class of optimization problems where some of the variables being selected are constrained to be integer-valued while some other can be real-valued (i.e., Mixed Integer (MI)), the objective function and/or some of the constraints are nonlinear functions (i.e., Nonlinear (NL)). In the context of optimization, “program” or “programming” (P) can be regarded as a synonym of optimization problem or optimization formulation. Notably, the class of MINLP problems is known to be computationally hard to solve (e.g., see [42]); therefore, exact solution of the problem is a nontrivial task, thus calling for approximation strategies to be put in place.

## Materials and methods

### Notation

We denote vectors by boldface lowercase letters and matrices with uppercase letters and we refer to the (*i*, *j*)-th entry of a matrix *A* by *A*_*ij*_. We represent by **0**_*n*_ and **1**_*n*_ vectors with *n* components, all equal to zero and to one, respectively. Moreover, we use 0_*n*×*m*_, 1_*n*×*m*_ to denote an *n*×*m* matrix with just zero and one entries, respectively. We use square brackets to denote the arguments of a function, e.g., we use *f*[*x*, *y*] to denote a function with arguments *x* and *y*. For the sake of brevity, where understood, we abbreviate a function of one or more arguments by *f*[⋅]. Given a vector $x\in R^{n}$ we denote by diag[***x***] the *n* × *n* diagonal matrix having diag[***x***]_*ii*_ = *x*_*i*_, for all *i* ∈ {1, …, *n*}. On the same footings, given a matrix $A\in R^{n}\times R^{n}$ we denote by diag[*A*] the *n* dimensional vector having diag[***A***]_*i*_ = *A*_*ii*_, for all *i* ∈ {1, …, *n*}. We denote by ⊙ the Hadamard (i.e., entry-wise) matrix product between matrices *A* and *B* with the same dimensions, i.e., the matrix *C* = *A* ⊙ *B* is such that *C*_*ij*_ = *A*_*ij*_ *B*_*ij*_; analogously, the Hadamard product between ***c*** = ***a*** ⊙ ***b*** between two vectors ***a***,***b*** is such that *c*_*i*_ = *a*_*i*_ *b*_*i*_. Remember that Hadamard product is commutative; moreover, notice that ***a*** ⊙ ***b*** = diag[***a***]***b*** = diag[***b***]***a***.

### SIR epidemics model

In this section we briefly review the SIR Epidemics model; the interested reader is referred to [35, 43] for further details. Let us consider a population of *N* individuals divided in *n* classes (e.g., by age or geographical area); we denote by *N*_*ℓ*_ the population in the *ℓ*-th class with $N=\sum_{\ell =1}^{n}N_{\ell }$. Moreover, let us indicate with *s*_*ℓ*_[*t*], *i*_*ℓ*_[*t*], *r*_*ℓ*_[*t*] the fraction of susceptible, infectious and removed individuals in the *ℓ*-th class at time *t* and with ***s***[*t*], ***i***[*t*], $r\left[t\right]\in R^{n}$ the stack of such variables for all classes. In the following, we assume that ***s***[0], ***i***[0], ***r***[0] ∈ [0, 1]^*n*^ and ***s***[0] + ***i***[0] + ***r***[0] = ***1***_*n*_. The SIR equations for such an heterogeneous population are given by

$$\begin{array}{c} \left\{ \begin{array}{c} \begin{array}{cc} \partial_{t}s\left[t\right] & =-s[t]⊙Bi[t]\\ \partial_{t}i\left[t\right] & =s[t]⊙Bi[t]-\gamma ⊙i[t]\\ \partial_{t}r\left[t\right] & =\gamma ⊙i[t], \end{array} \end{array}\right. \end{array}$$ (1)

where *B* is the *n* × *n* transmission matrix, *B*_*ij*_ being the rate at which a susceptible individual of class *i* meets an infectious individual of class *j* and becomes infected, while the vector $\gamma \in R^{n}$ collects the rates *γ*_*i*_ at which infectious individuals in the *i*-th class are removed from the infection cycle. Notably, the above choice for the initial conditions guarantees that ***s***[*t*], ***i***[*t*], ***r***[*t*] ∈ [0, 1]^*n*^ and ***s***[*t*] + ***i***[*t*] + ***r***[*t*] = ***1***_*n*_ for all time instants *t*.

#### End of the epidemics

Within the SIR model, an epidemic ends when ***i***[*t*] = **0**_*n*_, i.e., when

$$\begin{array}{c} s\left[t\right]+r\left[t\right]=1_{n}; \end{array}$$

such states are also called *end-of-epidemic* states. At this point, let us define ***n*** = [*N*_1_, …, *N*_*n*_]^*T*^ and let us consider the *overall* amount of infectious individuals *I*[*t*] = ***i***^*T*^[*t*]***n***; we have that

$$\begin{array}{c} \begin{array}{cc} \partial_{t}I\left[t\right] & =\partial_{t}i^{T}\left[t\right]n=i{\left[t\right]}^{T}(B^{T}\text{diag}\left[n\right]s\left[t\right]-\text{diag}\left[n\right]\gamma ). \end{array} \end{array}$$

In this view, since ***i***[*t*] ≥ 0, the total number of infected individuals *I*[*t*] is guaranteed to decrease with time *irrespective* of the particular value of ***i***[*t*] if and only if *B*^*T*^
diag[***n***]***s***[*t*] − diag[***n***]***γ*** < **0**_*n*_, i.e., if and only if [35, 44]

$$\begin{array}{c} Rs\leq 1_{n}, \end{array}$$ (2)

where $R=\text{diag}{\left(\gamma \right)}^{-1}\text{diag}{\left[n\right]}^{-1}B^{T}\text{diag}\left[n\right]$ is linked to the *next generation matrix* [43] that characterizes the stability of an end-of-epidemic state respect to infections and allows to calculate the *basic reproduction number* *R*_0_ indicating the theoretical rate of new infections that an infectious individual could generate.

#### Modeling assumptions and limits of the SIR model

The simplicity of the SIR model allows to design a scenario based on a limited number of parameters; it is thus one of the first models used to understand newborn epidemics. However, SIR models can sometime oversimplify the complex disease process. As an example, SIR models imply “full mixing”, i.e., the assumption that all individuals in the population are equally likely to be in contact with each other. To this respect, the heterogeneous SIR corrects such an issue by introducing classes and considering the heterogeneity in their contact rate. Also, we have employed a simplified SIR model with fixed populations, although in the original formulation it could account also for migration, births or deaths; such an approach is justified in the initial phase of an epidemic, where the time horizon is limited and variation in population can be disregarded. When an epidemic becomes endemic, SIR models can be easily extended to SIRS models where recovered individuals can become again susceptible. Furthermore, if there is a waiting time for an infected person to become infectious, SIR models can be extended to SEIR models by introducing an extra compartment E (i.e. “exposed”) that accounts for such an issue. In a “receding horizon” approach, where the model parameters are periodically adjusted to reflect the evolving knowledge on the epidemic, it is reasonable to resort to the SIR model during the first iteration, since its parameters are the simplest to estimate. Eventually, at later iterations, it is possible not only adjust the parameters, but also to switch to more sophisticated models (SEIR or even SEIRS if the situation becomes endemic) with the proceeding of time. Our framework easily allows to switch from SIR to SEIR or SEIRS model just by adding extra compartments; notice that, by defining by ***a***[*t*] = ***i***[*t*] + ***e***[*t*] the fraction of infected (i.e., exposed or infectious) individuals, the constrains ensuring the dampening of the epidemic for all classes (i.e., ∂***a***[*t*]<0) retain the same simple linear form of Eq (2), i.e., they depend only on the fraction of *infectious* individuals [44].

### Modeling interventions within the SIR model

In this section we modify the SIR model in order to explicitly account for possible interventions, namely, social distancing measures (e.g., adoption of personal protection equipment (PPE) and lockdowns) and vaccination. In view of later developments in the paper, it is convenient to first express the SIR model in discrete-time form. Notice that exact discretization of a nonlinear differential equation

$$\begin{array}{c} \partial_{t}x\left[t\right]=f\left[x\left[t\right]\right] \end{array}$$

with constant step size Δ*t* would be in the form

$$\begin{array}{c} x\left[\left(k+1\right)\Delta t\right]=x\left[k\Delta t\right]+\int_{k\Delta t}^{(k+1)\Delta t}f\left[x\left[t\right]\right]dt. \end{array}$$ (3)

However, given the complexity of the above exact method, it is convenient to consider an approximated relation. In particular, in this paper we resort to the Euler forward approximation, i.e., we set

$$\begin{array}{c} \partial_{t}x\left[t\right]{|}_{t=k\Delta t}\approx \frac{x[(k+1)\Delta t]-x[k\Delta t]}{\Delta t}; \end{array}$$

thus obtaining

$$\begin{array}{c} x[(k+1)\Delta t]\approx x[k\Delta t]+\Delta tf[x[k\Delta t]]. \end{array}$$

In other words, we approximate the continuous-time SIR model in Eq (1) via the following discrete-time equations

$$\begin{array}{c} \left\{ \begin{array}{c} \begin{array}{cc} s[(h+1)\Delta t] & =s[h\Delta t]-\Delta ts[h\Delta t]⊙Bi[h\Delta t]\\ i[(h+1)\Delta t] & =i[h\Delta t]+\Delta ts[(h+1)\Delta t]⊙Bi[h\Delta t]-\Delta t\gamma ⊙i[(h+1)\Delta t]\\ r[(h+1)\Delta t] & =r[h\Delta t]+\Delta t\gamma ⊙i[k]. \end{array} \end{array}\right. \end{array}$$ (4)

and we point out that, to avoid numerical instability as a result of the discretization, we choose Δ*t* = 0.01[*day*] for the parameters used in our case study (Other approaches allowing larger step size without causing instability could be considered, such as Euler backward integration, trapezoidal integration or Runge-Kutta methods; however, in this paper we opted for the Euler forward integration for the sake of simplicity).

Let us now incorporate two different types of intervention in the above discrete-time SIR model, accounting for the adoption of social distancing measures and for vaccination. Notably, in the following, we consider interventions such as vaccinations and social distancing measures that are planned at the level of the single day; as discussed next, such interventions will reflect in the discrete-time SIR model by assuming that the interventions remain constant over the day. As a consequence, such daily interventions will be indexed on a daily basis, while the variables in the discrete-time SIR model are indexed by *hΔt*. Moreover, we use the iterator *k* to denote the *k*-th day and, where needed, with a slight abuse of notation we use the iterator *k* to denote the value assumed by a variable of interest at the end of the *k*-th day, e.g., ***s***[*k*] = ***s***[*hΔt*], with *h* = *k*/Δ*t*, while we point out that the day corresponding to the time instant *hΔt* is given by ⌊*hΔt*⌋.

#### Social distancing interventions

In order to model the effect of social distancing measures in the SIR model, we observe that interventions such as lockdowns, limiting access to shops or imposing the adoption of PPEs, has the effect to reduce the rate at which susceptible individuals meet infectious individuals and/or become infected, modeled by the coefficients *B*_*ij*_. In order to model the effect of social distancing measures at the *k*-th day, let us define the *social distancing intensity* *E*_*ℓj*_[*k*] ∈ [0, *e*], where *e* is the maximum allowed intensity and *e* ≤ 1, as the intensity of the social distancing measures put in place for the *ℓ*-th and *j*-th class, e.g., *E*_*ℓj*_[*k*] = 0 means no measure is implemented, while *E*_*ℓj*_[*k*] = *e* means the maximum effort is spent in avoiding contacts between the *ℓ*-th and *j*-th classes. Notice that, by definition, we have that *E*_*ℓj*_[*k*] = *E*_*jℓ*_[*k*]. The above social distancing intensity coefficients account for the different strategies put in place. For instance, if the classes represent geographical regions, then a large value of *E*_*ℓj*_[*k*] implies large limitation of moving from the *ℓ*-th to the *j*-th one, while intermediate values of *E*_*ℓj*_[*k*] could be used to model a scenario where mobility is permitted for work and health circumstances. Conversely, if we consider age classes, then a situation where *E*_*ℓj*_[*k*] is large for all *j* could be used to model age-targeted lockdowns (e.g., for the elderly people).

As a result of the choice of *E*_*ℓj*_[*k*], we consider time-varying terms *B*_*ℓj*_[*k*] with the following structure

$$\begin{array}{c} B_{\ell j}\left[k\right]=B_{\ell j}\;(1-E_{\ell j}\left[k\right]) \end{array}$$ (5)

or, in a compact form

$$\begin{array}{c} B\left[k\right]=B⊙(1_{n\times n}-E\left[k\right]), \end{array}$$ (6)

where the *n* × *n* matrix *E*[*k*] collects the entries *E*_*ℓj*_[*k*]. In other words, *B*_*ℓj*_[*k*] corresponds to the nominal *B*_*ℓj*_ when no intervention is implemented and reaches zero in the case of a complete lockdown.

#### Vaccination

Let us now model the effect of vaccination on the discrete-time SIR model. In particular, we assume *m* different types of vaccines are available and we assume each vaccine *j* has an efficacy *η*_*j*_ ∈ [0, 1] after a single dose, while after the second dose the efficacy rises to *η*_*j*_ + Δ*η*_*j*_, with Δ*η*_*j*_ ∈ [0, 1 − *η*_*j*_]. Notice that the second dose is not required for all types of vaccines; we model this aspect by resorting to a coefficient

$$\begin{array}{c} \phi_{j}=\{\begin{array}{cc} 1, & \;\text{if}\;\text{the}\;\text{second}\;\text{dose}\;\text{is}\;\text{required}\;\text{for}\;\text{the}\;j\text{-th}\;\text{vaccine}\\ 0, & \;\text{otherwise}. \end{array} \end{array}$$

Moreover, for each type *j* of vaccine we assume a time window of $\tau_{j}^{I}$ days is required for the vaccine to take effect after the first dose, while we use $\chi_{j}\geq \tau_{j}^{I}$ to denote the time window between the first and the second dose (if required) and $\tau_{j}^{II}$ to denote the time window between the administration of the second dose and the reach of complete effect. In other words, an administration of vaccine *j* on the *k*-th day has an initial effect on day $k+\tau_{j}^{I}$ and a complete effect on day $k+\chi_{j}+\tau_{j}^{II}$, while *τ*^*I*^, *τ*^*II*^ are the times estimated from pharmacological trials during which vaccinated individuals are still exposed to the infection.

In order to model the effect of vaccination, let us indicate with *X*_*ℓj*_[*k*] the units of first doses of vaccines of the *j*-th type that are injected to the *ℓ*-th class of population at the *k*-th day and let $X\left[k\right]\in N^{n\times m}$ be the matrix with integer entries collecting such variables. Moreover, let *Y*_*ℓj*_[*k*] denote the amount of units of second dose of vaccines of the *j*-th type that are injected to the *ℓ*-th class of population at the *k*-th day.

In this view, the contribution Δ*r*_*ℓ*_[*k*] at day *k* to the number of removed individuals belonging to the *ℓ*-th class as a result of vaccination satisfies

$$\begin{array}{c} N_{\ell }\Delta r_{\ell }\left[k\right]=\sum_{j=1}^{m}X_{\ell j}\left[k-\tau_{j}^{I}\right]\eta_{j}+\sum_{j=1}^{m}Y_{\ell j}\left[k-\tau_{j}^{II}\right]\Delta \eta_{j}; \end{array}$$

i.e., the contribution of the *j*-th vaccine to Δ*r*_*ℓ*_[*k*] corresponds to the fraction of individuals that were vaccinated $\tau_{j}^{I}$ days before with the first dose of the *j*-th vaccine, weighted by its efficacy *η*_*j*_, plus the fraction of individuals that were vaccinated $\tau_{j}^{II}$ days before with the second dose of the *j*-th vaccine, weighted by the residual efficacy Δ*η*_*j*_. In other words, Δ*r*_*ℓ*_[*k*] is given by

$$\begin{array}{c} \Delta r_{\ell }\left[k\right]=\frac{1}{N_{\ell }}(\sum_{j=1}^{m}X_{\ell j}\left[k-\tau_{j}^{I}\right]\eta_{j}+\sum_{j=1}^{m}Y_{\ell j}\left[k-\tau_{j}^{II}\right]\Delta \eta_{j}) \end{array}$$ (7)

where, for the sake of consistency, we assume *X*[⋅], *Y*[⋅] are zero when their argument is negative.

Finally, we assume that

$$\begin{array}{c} Y_{\ell j}\left[k\right]\leq \phi_{j}X_{\ell j}\left[k-\chi_{j}\right], \end{array}$$ (8)

i.e., if required (*ϕ*_*j*_ = 1), the units of second dose of the *j*-th type of vaccine injected at the *k*-th day must not trespass the units of first dose injected *χ*_*j*_ days before; otherwise (*ϕ*_*j*_ = 0), no second dose is considered. Notice that, being Eq (8) an inequality, the second dose is not mandatory, and it is possible to implement policies where only a fraction of the population receiving the first dose receives also the second as suggested by the UK study SIREN [45].

#### Resulting SIR model

To conclude the section, let us show the expression of the discrete-time SIR model where the above interventions are explicitly considered. In particular, as a result of the social distancing intervention, matrix *B* is replaced by the matrix *B*[*k*] in Eq (6); moreover, in order to take into account the effect of vaccination, we assume Δ***r***[*k*] is subtracted at each day *k* from the fraction of susceptible individuals, and is simultaneously added to the removed ones, without influencing the fraction of infectious individuals. We reiterate that the effect of the interventions at day *k* is mediated by the sampling time Δ*t*; in other words, the discrete-time SIR model becomes

$$\begin{array}{c} \left\{ \begin{array}{c} \begin{array}{cc} s[(h+1)\Delta t] & =s[h\Delta t]-\Delta ts[h\Delta t]⊙B[k]i[h\Delta t]-\Delta t\Delta r[k]\\ i[(h+1)\Delta t] & =i[h\Delta t]+\Delta ts[(h+1)\Delta t]⊙B[k]i[h\Delta t]-\Delta t\gamma ⊙i[(h+1)\Delta t]\\ r[(h+1)\Delta t] & =r[h\Delta t]+\Delta t\gamma ⊙i[k]+\Delta t\Delta r[k] \end{array} \end{array}\right. \end{array}$$ (9)

or, in a compact form

$$\begin{array}{c} z\left[\left(h+1\right)\Delta t\right]=f(z[h\Delta t],\Delta r[k],E[k]),\;k=\left\lfloor h\Delta t\right\rfloor , \end{array}$$ (10)

where ***z***[⋅] = [***s***^*T*^[⋅], ***i***^*T*^[⋅], ***r***^*T*^[⋅]]^*T*^.

### Optimization formulation

The above SIR model with explicit intervention terms is the natural cornerstone for the planning of such interventions.

In particular, we assume a finite-time horizon of *k*_max_ days and we consider a scenario where at the 0-th day the epidemics is described by given initial conditions ***s***[0], ***i***[0], ***r***[0] ∈ [0, 1]^*n*^ with ***s***[0] + ***i***[0] + ***r***[0] = ***1***_*n*_.

The aim of the proposed formulation is to plan the different interventions to be put in place to guarantee the reach of the end of the epidemics on day *k*_max_, i.e., we want to enforce dynamical constraints that represent the evolution of the proposed variation of the SIR model (Eq (9), with *B*[*k*] and Δ*r*_*i*_[*k*] defined as in Eqs (6) and (7), respectively), together with the requirement that the SIR model reaches the herd immunity surface. The latter requirement is equivalent to enforcing a constraint in the form

$$\begin{array}{c} s\left[k_{\text{max}}\right]+r\left[k_{\text{max}}\right]=1_{n}, \end{array}$$ (11)

ensuring that an end-of-epidemic state is reached; at the same time, we want to guarantee that new infections die out. This requirement, as discussed above, is equivalent to enforcing a constraint in the form of Eq (2). Notably, *B*[*k*] is time varying; however, when the final planning instant *k*_max_ is reached, it is reasonable to assume that non-pharmaceutical interventions are discontinued and *E*[*k*_max_] = 0_*n* × *n*_; thus, the conditions for avoiding epidemic overburst is

$$\begin{array}{c} Rs\left[k_{\text{max}}\right]\leq 1_{n}. \end{array}$$ (12)

Let us now discuss the choice variables of the proposed model; specifically, the model aims at identifying the units *X*[*k*]≥0_*n* × *n*_ and *Y*[*k*] ≥ 0_*n* × *n*_ of first and second doses of vaccine to be injected on the *k*-th day and the intensity of the social distancing measures *E*[*k*] ∈ [0, *e*]^*n* × *n*^ on the *k*-th day, for all *k* ∈ {1, …, *k*_max_}. Notice that, as discussed above, the latter variables must satisfy

$$\begin{array}{c} E\left[k\right]=E^{T}\left[k\right],\;\forall k\leq k_{\text{max}}. \end{array}$$ (13)

Let us now focus on aspects related to vaccination. In order to plan for such intervention, we consider a situation where vaccines become available in batches. Specifically, we assume there are specific days $k_{1},\ldots ,k_{w_{\text{max}}}$ in which batches of vaccines are received, and we use $q\left[k\right]\in R^{m}$ to denote the vector collecting the total units of vaccines received as of day *k* for each type of vaccine.

In order to guarantee that the vaccination plan is sound, we need to impose that the cumulative units of vaccine that are injected as of day *k* do not trespass the received ones, for each type, i.e.,

$$\begin{array}{c} \sum_{h=0}^{k}{\left(X[h]+Y[h]\right)}^{T}1_{n}\leq q\left[k\right],\;\forall k. \end{array}$$ (14)

Notice that, in order to guarantee that second doses do not trespass the first ones, we consider the constraint in Eq (8); moreover, to guarantee that the overall amount of doses does not exceed the population in each class, we consider a constraint in the form

$$\begin{array}{c} \sum_{k=1}^{k_{\text{max}}}(X[k]+Y[k])1_{m}\leq n. \end{array}$$ (15)

Finally, let us assume that a maximum overall number *l*_*h*_ of daily inpatient beds, *l*_*sh*_ of which being intensive care inpatient beds, are available. In this view, in order to enforce that the amount of hospitalizations and intensive care hospitalizations do not overcome the limits, we consider constraints in the form

$$\begin{array}{c} \sigma_{h}^{T}\text{diag}\left[n\right]i\left[k\right]\leq l_{h},\;\forall k\leq k_{\text{max}} \end{array}$$

and

$$\begin{array}{c} \sigma_{sh}^{T}\text{diag}\left[n\right]i\left[k\right]\leq l_{sh},\;\forall k\leq k_{\text{max}}, \end{array}$$

where the vectors ***σ***_*h*_ and ***σ***_*sh*_ collect the hospitalization and severe hospitalization rates for each class, respectively, and diag[***n***]***i***[*k*] is the vector collecting the population of infectious individuals in each class.

Let us now discuss the objective function of the proposed formulation. In particular, we aim to minimize the cumulative intensity of the the social distancing measures over the considered time horizon, i.e.,

$$\begin{array}{c} \sum_{k=0}^{k_{\text{max}}}\sum_{\ell =1}^{n}\sum_{j=1}^{n}E_{\ell j}\left[k\right]. \end{array}$$

Notice that, within any optimization formulation, minimizing the objective function is secondary to constraint satisfaction. Therefore, within the proposed formulation, reaching of the herd immunity and avoiding the collapse of the healthcare system represent a priority with respect to the minimization of the overall intensity of the social intervention. In other words, solutions that have small objective function value but violate the constraints will be deemed *unfeasible* and will be discarded by any solver. Overall, the proposed formulation consists of the following *Mixed Integer Nonlinear Programming* (MINLP) problem.

$$\begin{array}{c} \begin{array}{cc} & \min\sum_{k=0}^{k_{\text{max}}}\sum_{\ell =1}^{n}\sum_{j=1}^{n}E_{\ell j}\left[k\right]\\ & \text{subject}\;\text{to}\\ \begin{array}{c} \left(I\right)\\ \left(II\right)\\ \left(III\right)\\ \left(IV\right)\\ \left(V\right)\\ \left(VI\right)\\ \left(VII\right)\\ \left(VIII\right)\\ \left(IX\right)\\ \left(X\right)\\ \left(XI\right)\\ \left(XII\right)\\ \left(XIII\right)\\ \left(XIV\right)\\ \left(XV\right) \end{array} & \left\{ \begin{array}{cc} z\left[\left(h+1\right)\Delta t\right]=f(z[h\Delta t],\Delta r[k],E[k]),\;\;\forall h\leq k_{\text{max}}/\Delta t\\ B\left[k\right]=B⊙\left(1_{n\times n}-E\left[k\right]\right),\;\;\forall k\leq k_{\text{max}}\\ \Delta r_{\ell }\left[k\right]=\frac{\sum_{j=1}^{m}X_{\ell j}\left[k-\tau_{j}^{I}\right]\eta_{j}+\sum_{j=1}^{m}Y_{\ell j}\left[k-\tau_{j}^{II}\right]\Delta \eta_{j}}{N_{\ell }},\;\;\forall \ell \;\leq \;n,\;k\;\leq \;k_{\text{max}}\;\;\\ s\left[k_{\text{max}}\right]+r\left[k_{\text{max}}\right]=1_{n},\\ Rs\left[k_{\text{max}}\right]\leq 1_{n}, & \\ \sum_{h=0}^{k}{\left(X[h]+Y[h]\right)}^{T}1_{n}\leq q\left[k\right],\;\;\forall k\leq k_{\text{max}}\\ \sum_{k=0}^{k_{\text{max}}}(X[k]+Y[k])1_{m}\leq n,\\ Y_{\ell j}\left[k\right]\leq \phi_{j}X_{\ell j}\left[k-\chi_{j}\right],\;\;\forall k\leq k_{\text{max}},\;\ell \leq n,\;j\leq m\\ E\left[k\right]=E^{T}\left[k\right],\;\;\forall k\leq k_{\text{max}}\\ \sigma_{h}^{T}\text{diag}\left[n\right]i\left[k\right]\leq l_{h},\;\;\forall k\leq k_{\text{max}}\\ \sigma_{sh}^{T}\text{diag}\left[n\right]i\left[k\right]\leq l_{sh},\;\;\forall k\leq k_{\text{max}}\\ r\left[k\right],s\left[k\right],i\left[k\right]\in {\left[0,1\right]}^{n},\;\;\forall k\leq k_{\text{max}}\\ E\left[k\right]\in {\left[0,e\right]}^{n\times n},\;\;\forall k\leq k_{\text{max}}\\ X\left[k\right],Y\left[k\right]\in R_{\geq 0}^{n\times m},\;\;\forall k\leq k_{\text{max}}\\ X\left[k\right],Y\left[k\right]\;\text{integer}\;,\;\;\forall k\leq k_{\text{max}}. \end{array}\right. \end{array} \end{array}$$ (16)

In other words, constraints (I)–(III) model the requirement that the fraction of susceptible, infectious and removed individuals evolve according to the proposed SIR model accounting for the interventions in terms of social distancing and vaccination. Constraint (IV) accounts for reaching an end-of-epidemic state, while Constraint (V) guarantees that new infections die out. Constraint (VI) and (VII) guarantee that the amount of used doses of vaccine do not overcome the available ones or the overall population, respectively. Constraint (VIII) enforces that the second doses (if required) are injected after the adequate time window. Constraint (IX) prescribes that *E*(*k*) is symmetric, thus implying that the social distancing effort reducing the influence of the *i*-th class on the *j*-th one has a specular effect on the influence of the *j*-th class on the *i*-th one. Constraints (X) and (XI) guarantee that the regular and intensive care hospitalizations do not overcome the maximum limit. Finally, constraints (XII)–(XV) guarantee the well-posedness of the variables considered in the formulation.

#### Approximation strategy

Notice that the proposed formulation amounts to a Mixed Integer Nonlinear Programming problem. In particular, we observe that the model requires a nontrivial amount of variables and constraints (i.e., *O*(max{*n*/Δ*t*, *n*^2^, *nm*}) for each day of planning. Moreover, we observe that the problem is nonconvex (i.e., considering the nonlinear equality constraints corresponding to the SIR model as two inequality constraints, there is no way both are convex). However, since the units of vaccines involved in the planning are expected to be large, it makes sense to attempt to reduce complexity by considering a continuous relaxation, i.e., dropping integrity constraints. However, also in the case of a convex objective function and a continuous relaxation, the problem has high chances to be NP-Hard (e.g., see [46, 47]), thus calling for approximated solutions.

In this paper, our strategy to calculate an approximated solution is to resort to an approximated solver. In fact, we observe that ***s***[⋅], ***i***[⋅], ***r***[⋅], Δ***r***[⋅] are actually functions of the variables *E*[*k*], *X*[*k*], *Y*[*k*], even though it is nontrivial to express this dependency in a closed form. Therefore, our strategy is to consider only the variables *E*[*k*], *X*[*k*], *Y*[*k*] and to express the constraints and the objective function in an algorithmic way, resorting to an approximated solver. Specifically, we use the MIDACO optimization software which implements an extension of the evolutionary Ant Colony Optimization meta-heuristic [48] and which has been developed especially for highly non-linear real-world applications. See [49, 50] for a focus of the performance of MIDACO software with respect to the state of the art.

Note that the suggested strategy is independent of a particular solver, but the non-convex nature of the optimization problem suggests an evolutionary approach, like genetic algorithms [51]. Furthermore, the dimensionality of the resulting MINLP in the next case study is very large-scale, consisting of 76650 decision variables and 18295 constraints, and therefore requires a solver that can handle such dimensionality. Finally, we point out that, since in our implementation we chose to evaluate the variables ***s***[⋅], ***i***[⋅], ***r***[⋅], Δ***r***[⋅] as a function of the variables *E*[*k*], *X*[*k*], *Y*[*k*], a positive consequence is that the step size Δ*t* used to discretize the SIR model has no effect on the overall number of choice variables, which is one of the major sources of complexity for the solution of MINLP formulations (e.g., see [52]).

### Computational setting

The optimization with MIDACO was conducted on an Intel^®^Xeon^®^CPU E7 2860 @ 2.27GHz. The CPU runtime for the optimization was fixed to five days. All MIDACO parameters were used by their default values, that means that a feasiblity accuracy of 0.001 was used for all individual constraints listed in Eq (16).

## Results

In this section, we test the effectiveness of the proposed formulation by considering a case study with realistic parameters consistent with the current COVID-19 pandemics and relative vaccines. Specifically, we focus on Italy and we identify the optimal vaccination policies over a one-year time horizon, considering 15 age classes (see Table 1) and three types of vaccines. Specifically, Table 2 reports the information regarding the efficacy *η*_*j*_, the delay required to appreciate the effect of the first dose $\tau_{j}^{I}$, the delay between doses *χ*_*j*_, the efficacy of the second dose Δ*η*_*j*_ and the delay required to appreciate the effect of the second dose $\tau_{j}^{II}$. The fictional vaccines considered mimic real vaccines, and the parameters are estimates based on data in [53–55]. Notably, we assume that the three considered types of vaccine are available only in batches, at specific time instants and in limited amount for each batch, as summarized in Table 3. For simplicity, it is assumed that at regime batches reach between six million and nine million of doses per trimester; such figures are consistent with what has been planned and deployed in Italy [56].

**Table 1:** Population in the different age classes (Source: [60]). COVID-19 hospitalization, severe hospitalization and death rates as of April 2021 (source: [59]).

Age Class	Population	Hospitalization	Severe Hospitalization	Death
00–04	2645566	10.7%	0.31%	0.10%
05–09	2769974	10.7%	0.31%	0.10%
10–14	2932459	5.81%	0.23%	0.10%
15–19	2968742	5.81%	0.23%	0.10%
20–24	3041263	6.28%	0.32%	0.10%
25–29	3281737	6.28%	0.32%	0.10%
30–34	3531873	8.84%	0.77%	0.14%
35–39	3877837	8.84%	0.77%	0.14%
40–44	4387315	11.97%	1.91%	0.26%
45–49	5060898	11.97%	1.91%	0.26%
50–54	5068741	16.86%	3.59%	0.57%
55–59	4869741	16.86%	3.59%	0.57%
60–64	4102571	27.33%	6.79%	2.73%
65–69	3554615	27.33%	6.79%	2.73%
70+	10297032	34.70%	3.27%	14.80%

**Table 2:** Efficacy of the vaccines considered in the proposed case study. Vaccine A mimics BNT162b2 (Pfizer & BioNTech); vaccine B mRNA-1273 (Moderna) and vaccine C ChAdOx1 nCoV-2019 (University of Oxford/AstraZeneca). The source for the estimates are: [53–55].

	Vaccine A	Vaccine B	Vaccine C
**Efficacy** (1st dose) *η*_*j*_	0.89	0.89	0.70
**Delay for effect** (1st dose) $\tau_{j}^{I}$ [*days*]	15	15	15
**Delay between doses** *χ*_*j*_ [*days*]	28	28	84
**Efficacy** (2nd dose) Δ*η*_*j*_	0.06	0.05	0.20
**Delay for effect** (2nd dose) $\tau_{j}^{II}$ [*days*]	15	15	15

**Table 3:** Units of Vaccines of each type that are assumed to become available in batches at specific days.

Day	Vaccine A	Vaccine B	Vaccine C
0	500000	500000	500000
30	500000	500000	500000
60	1000000	1000000	1000000
90	2000000	2000000	2000000
120	2000000	2000000	2000000
150	2000000	2000000	2000000
180	2000000	2000000	2000000
210	2000000	2000000	2000000
240	2000000	2000000	2000000
270	3000000	3000000	3000000
300	3000000	3000000	3000000
330	3000000	3000000	3000000

Moreover, we consider a scenario where only a small fraction (i.e., 0.01%) of the age class in thee range 35 − 39 years is initially infected and we assume the maximum daily inpatient beds are *l*_*h*_ = 1000, while the maximum daily intensive care inpatient beds are *l*_*sh*_ = 100.

Notice that, for the sake of simplicity, we allow vaccination for all age groups, even though Italian regulation does not yet allow COVID-19 vaccination under the age of 5.

### Parameter tuning

**Fig 1:**
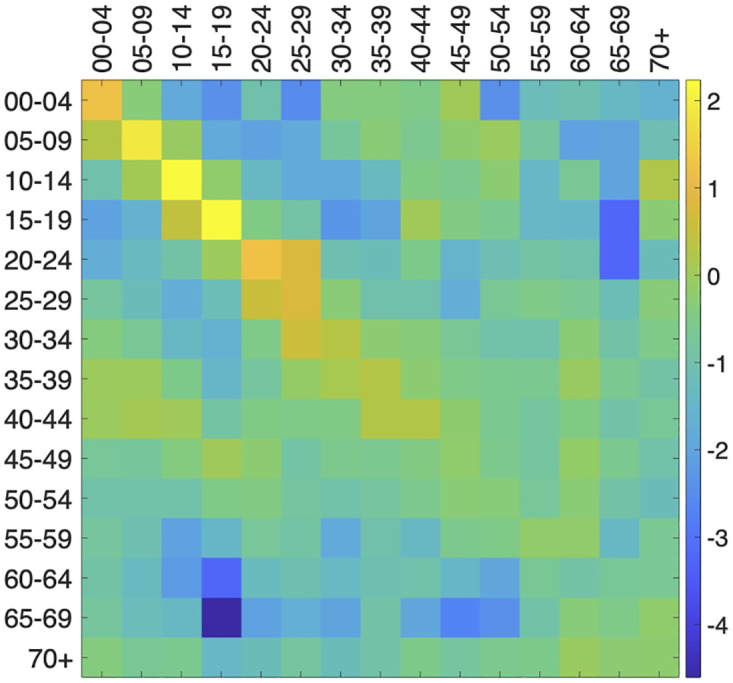
Elements of the matrix *K* of physical contacts among age classes in Italy (source: [57]). For the sake of readability, the colors of the cells corresponds to ln(*K*_*ij*_).

In order to tune our formulation, we consider the country contact matrix *K* (see Fig 1), as estimated in [57] for Italy. In particular, only physical contacts have been considered. Notice that the element *K*_*ij*_ of a contact matrix from [57] can be considered proportional to the probability that an individual in the *i*-th age class meets an individual in the *j*-th; thus, *B* = Λ ⊙ *K* where Λ_*ij*_ is the probability that a contact between *i* and *j* results in an infection. In this case study, we will use a constant Λ_*ij*_ = λ; analogously, we will use a constant *γ*.

To fix the parameters, we will consider a basic reproduction number (i.e., the potential number of new infected generated by one case) *R*_0_ = 3—a value that has been estimated for COVID19 in France [58]. Since for an heterogeneous compartmental model of the form of Eq (1) the role of the basic reproduction number is played by ∥R∥ [22, 43], we can rescale λ to obtain a basic reproduction number equivalent to the observed one:

$$\begin{array}{c} \lambda =\frac{\gamma R_{0}}{∥K∥}, \end{array}$$

i.e.,

$$\begin{array}{c} ∥R∥=∥\lambda \;\text{diag}{\left(\gamma \right)}^{-1}\text{diag}{\left(n\right)}^{-1}K^{T}\text{diag}\left(n\right)∥=R_{0}. \end{array}$$ (17)

The other parameters required to tune the proposed model are the rates of hospitalization, of severe hospitalization, and of death; such parameters can be found in the ECDC ninth risk assessment update for COVID-19 in the EU/EEA and the UK [59] and are reported in Table 1.

### Experimental results

**Fig 2:**
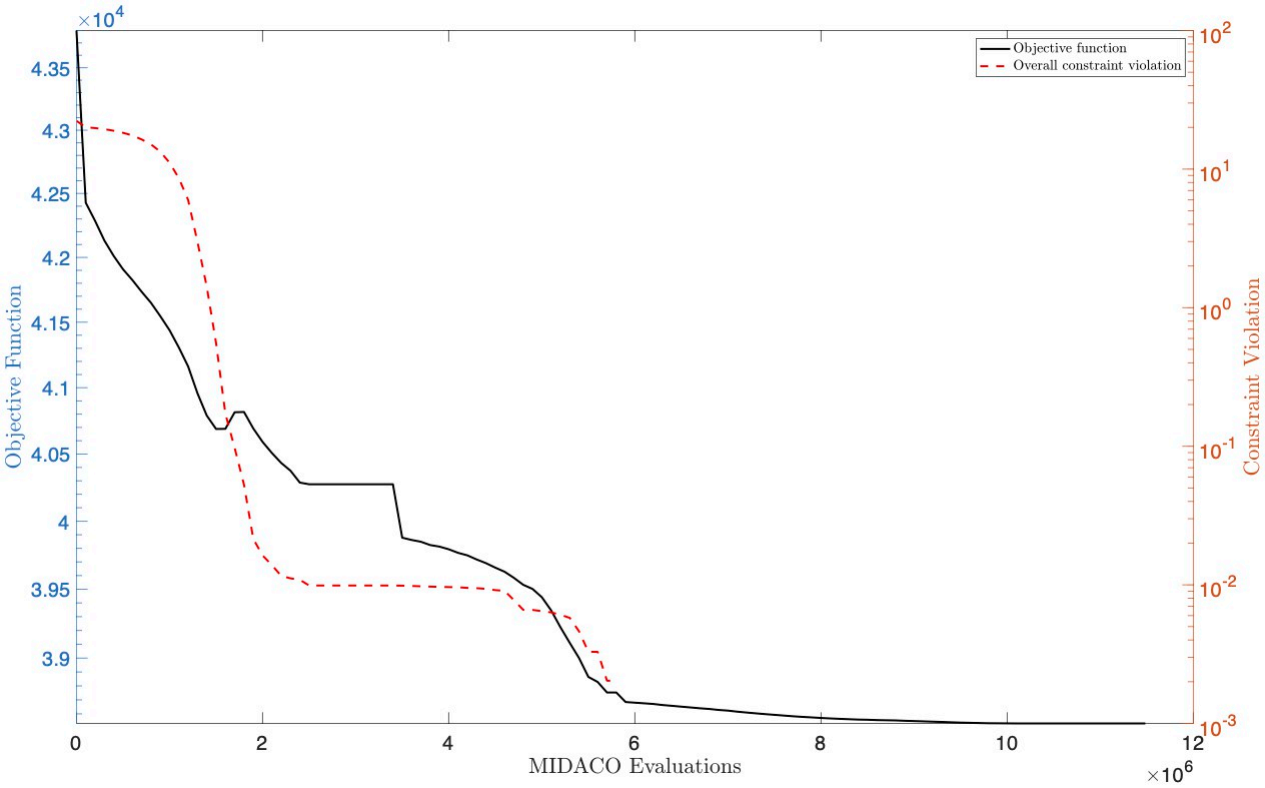
Objective function value and overall constraint violation for the solution found using MIDACO, plotted against the number of candidate solutions evaluated.

Let us now discuss the experimental results from the computational point of view. Fig 2 shows the results of MIDACO in terms of objective function value and overall violation of the constraints, plotted against the number of candidate solutions evaluated by MIDACO. As shown by the figure, we observe that a feasible solution is obtained in about 6 × 10^6^ evaluations. As for the objective function, we observe that while the solution is infeasible there is a relevant reduction over time; in particular, we reach a steady solution after about 9 × 10^6^ evaluations. Overall, these results suggest the reach of a local minimum.

Having discussed the computational aspects, let us now focus on the structure of the found solution.

**Fig 3:**
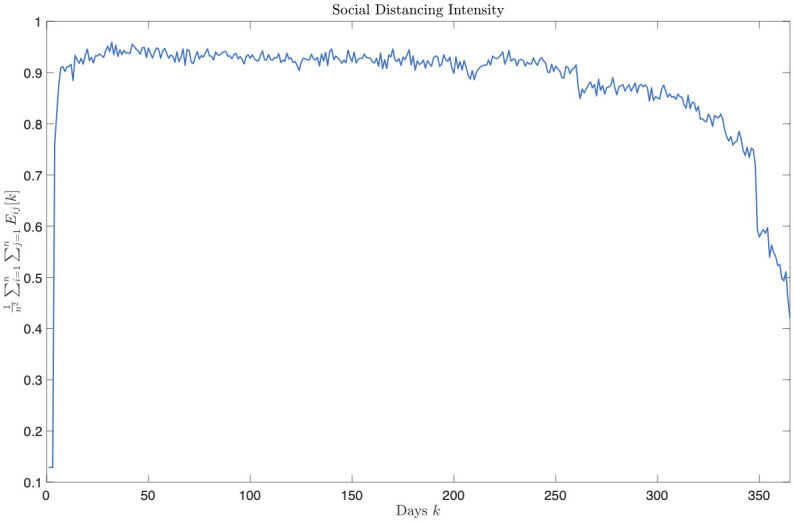
Intervention plan corresponding to the found solution in terms of the intensity of social distancing measures plotted against time.

**Fig 4:**
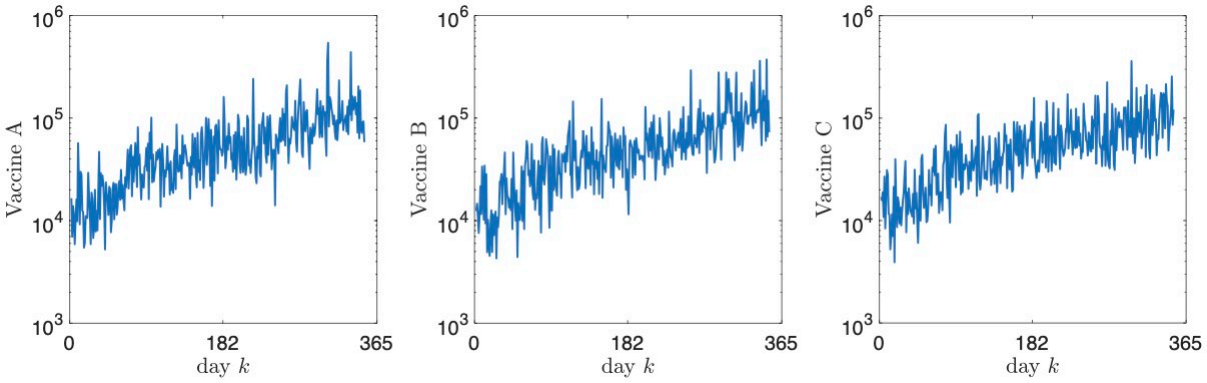
Intervention plan corresponding to the found solution in terms of the units of the different types administered for each day.

**Fig 5:**
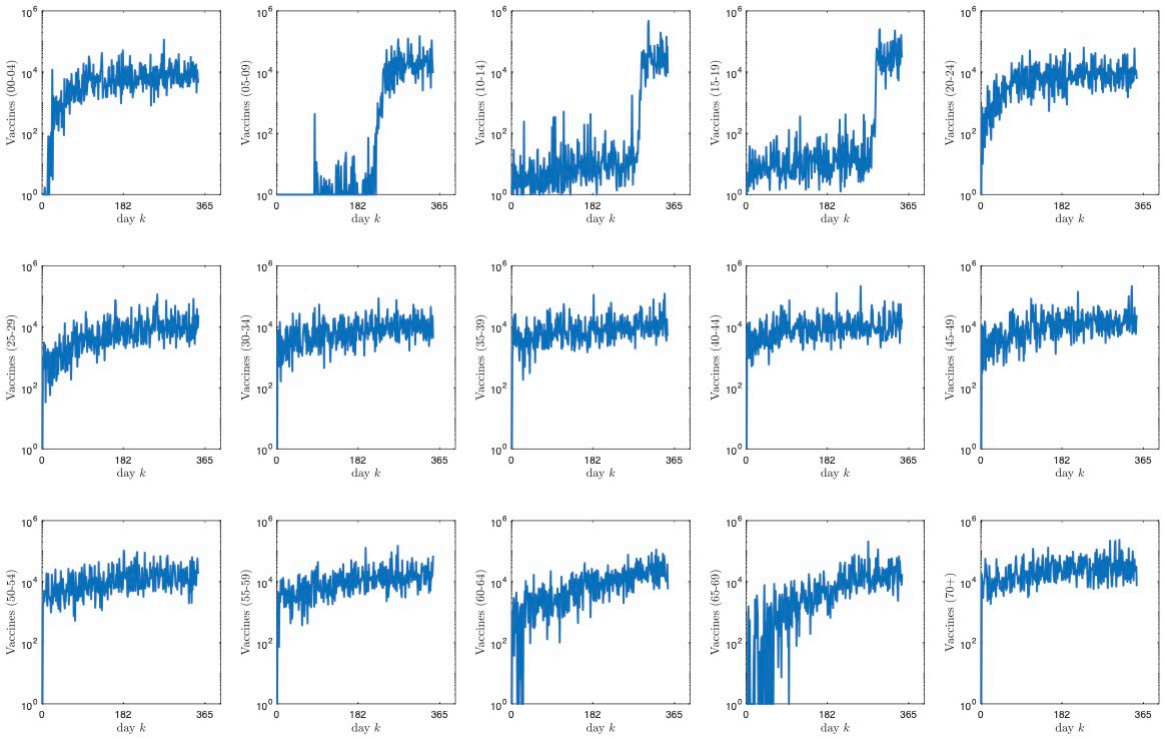
Intervention plan corresponding to the found solution in terms of the units of vaccines administered to the different age classes for each day.

**Fig 6:**
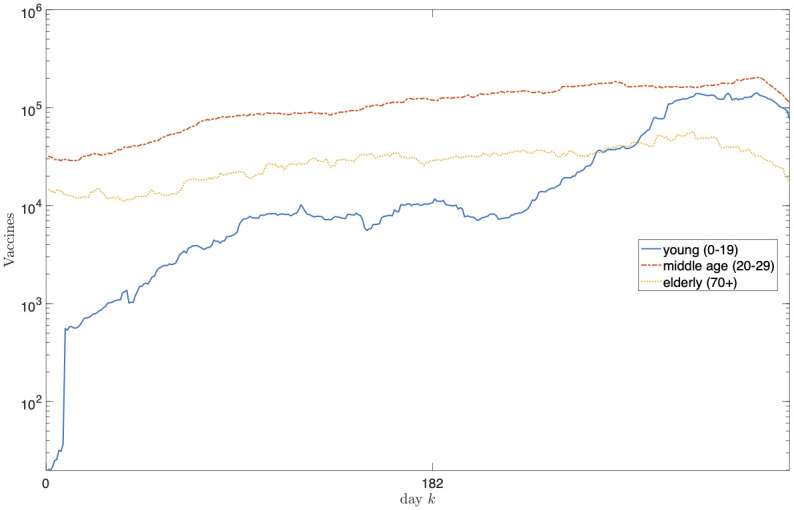
Intervention plan corresponding to the found solution in terms of the units of vaccines administered to the different age classes for each day. The plot aggregates the age classes into the *young* (0–19 years), *middle-age* (19–69 years) and *elderly* (≥70 years) macro-classes. To improve readability, data has been smoothed using a 30-day moving average filter.

Figs 3–6 report the structure of the interventions encoded by the found solution. Specifically, according to Fig 3, it can be noted that the the social distancing measures are initially quite intense, and only at the end of the planning horizon there is a partial reduction. Fig 4 shows how vaccine usage is distributed based on the type of vaccine. According to the figure, there is no noticeable difference; this is likely the effect of the scarcity of vaccines in our scenario. Moreover, Fig 5 (as well as Fig 6, where the same data is aggregated and smoothed to improve readability) shows that, in the early stages of the planning, due to the scarcity of vaccines, there is a preference for vaccinating individuals in the age range 20–69 years over young and elderly people; notably, such an age range receives a more or less steady amount of vaccines over time. This stems from the fact that, as discussed above, in the proposed formulation the objective of minimizing the intensity of the social distancing is secondary to constraint satisfaction, i.e., less restrictive social distancing measures can be considered only if they allow the reach the herd immunity and prevent the collapse of the healthcare system. In other words, candidate solutions where strict social distancing was released earlier than the identified solution have been discarded by the solver due to some violation of the constraints.

**Fig 7:**
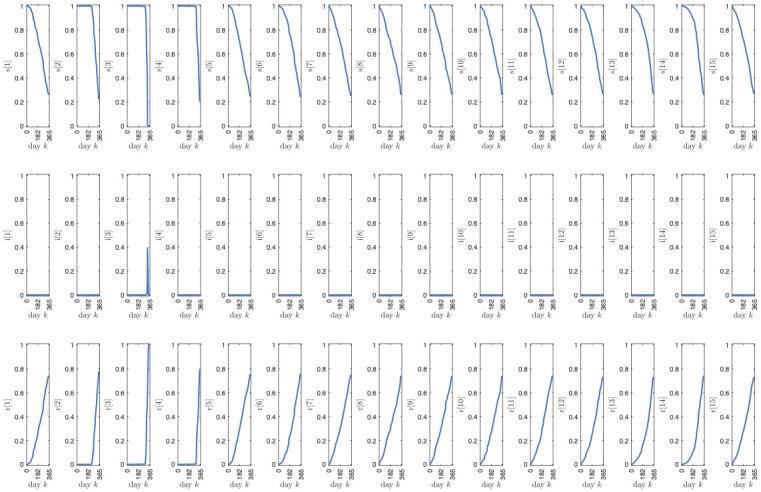
Evolution of the proposed SIR model accounting for the effect of social distancing and vaccines, based on the found solution. The first, second and third row of plots correspond to the fraction of susceptible, infected and removed individuals, respectively, while the *k*-th column of plots corresponds to the *k*-th age class.

Fig 7 shows the evolution of the proposed SIR model accounting for the effect of social distancing and vaccines, as a result of the interventions planned within the found solution. It can be noted that, due to the strict social distancing measures, only a small fraction of the population becomes infected, with a noticeable peak for the age class in the range 10 − 14 years in correspondence to the softening of the lockdown measures (i.e., around day *k* = 320). Notice that the fraction of susceptible individuals is slowly eroded due to vaccination, while the fraction of removed has a consequent slow growth due to the resulting immunization.

**Fig 8:**
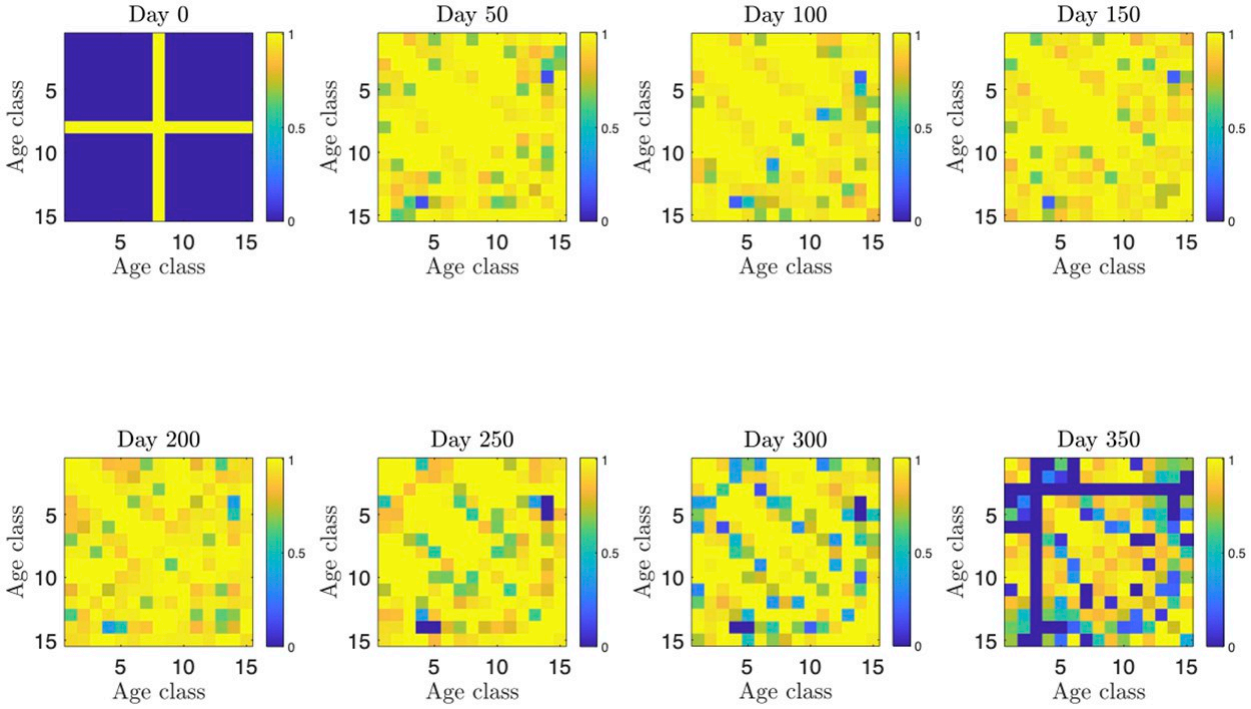
Intensity of lockdown within the found solution for the different age classes and for selected days over the considered time horizon. The intensity is shown with a blue to yellow scale, where blue represents no social distancing and yellow a complete lockdown.

Fig 8 breaks down the social distancing measures by age class; it can be noted that most of the considered time horizon all age classes are strongly restrained in their interaction. Then, around the end of the planning horizon the lockdown is significantly lifted for the age class in the range 10–14 years (from which the peak in the infected fraction of this age class).

**Fig 9:**
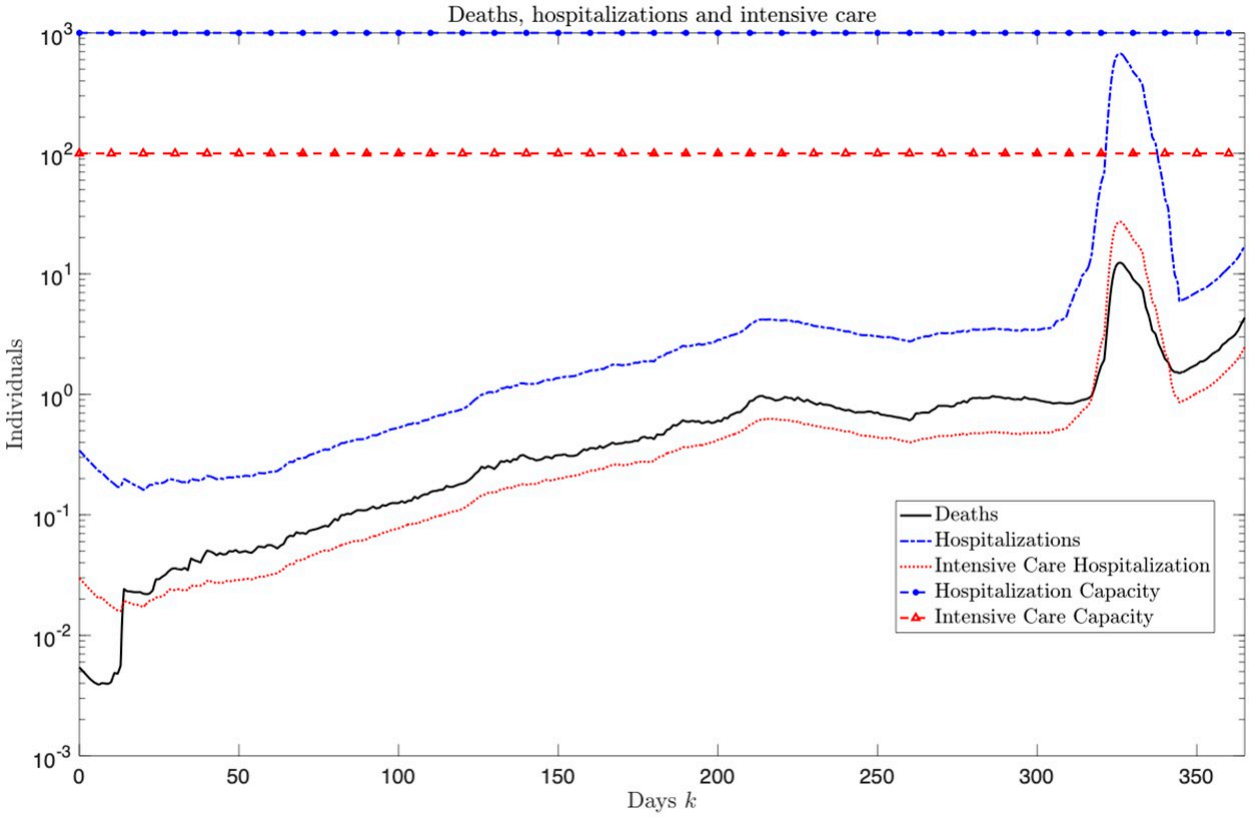
Deaths, hospitalizations and intensive care occupancies corresponding to the found solutions, plotted for each day.

Finally, Fig 9 shows the results of the planning in terms of deaths, hospitalization and intensive care occupancy. It can be noted that the solution found corresponds to a situation where the capacity in terms of regular and intensive care beds is not reached, thus avoiding the collapse of the healthcare system.

## Conclusion

In this paper we develop a fine-grained model to support the plan for intervention in order to contrast newborn epidemics. The proposed approach is particularly suitable for infections like the ongoing COVID-19 epidemic, characterized by high infection rate and able to pose the healthcare system and society under stress in terms of intensive care occupancy, deaths or economic consequences. Moreover, the approach allows to plan interventions that blend large-scale non-pharmaceutical interventions along with the pharmaceutical ones. Specifically, we build up the planning on two two main types of intervention, namely, non-pharmaceutical (essentially social distancing measures) and vaccination. In order to model the effect of such interventions, we develop a variation of the SIR epidemics model with heterogeneous population; specifically, we assume social distancing intensity to reflect into a reduction of the infection rates, while vaccination to have a partial and delayed immunization effect. Notably, we consider several population classes, several vaccines with different efficacy and with partial and delayed effect, the possibility of a second dose, the availability of vaccines in batches, the need of reaching the herd immunity and the requirement to avoid congestions in the healthcare system. Interestingly, besides representing a detailed, day-to-day planning, the proposed approach also provides useful insights from the clinical and policy-making point of view. In fact, the plan identified by the proposed methodology suggests that, initially, the scarcity of vaccines should be faced by enforcing a strict social distancing, and that vaccination priority should be given to the elderly and “middle-age” population over the younger one. The proposed model exhibits a nontrivial degree of complexity, and the identification of efficient approximated ways to solve it represents a challenging task. Yet, the proposed model represents a remarkably descriptive framework, and future work will be mainly devoted to incorporate other important perspectives for policy and decision makers, such as geographical [61], economical [62] and logistic aspects [63], social equity in the vaccine distribution [64], and skepticism of the population towards vaccines [65].

A last envisaged research perspective is related to the time duration of the planning. In fact, due to the change of the overall epidemiological or pharmaceutical situation, a yearly time horizon could be deemed excessively long; yet, the reach of the herd immunity requires a sufficiently wide time frame. To this end, a viable future work direction is to adopt a “receding horizon” perspective [41, 66], where the model is updated after a given period of time (e.g., one month or three months) and a new planning is executed starting from the epidemiological situation at that time. In particular, as new evidence regarding the prevalence of new strains is gathered, the model could be updated (e.g., changing the basic reproduction number [67, 68], adding new compartments such as the fraction of asymptomatic individuals [69, 70], considering re-infections [71], etc.). Moreover, as new vaccines are developed (or discontinued, as in the case of the Vaxzevria vaccine in Italy [72]) and their effectiveness is better assessed with respect to the circulating variants of the virus, the model can be updated accordingly (e.g., requirement of a booster dose, change in effectiveness, change in the time between doses, etc.). In other words, while the proposed methodology could be considered an *open loop* approach, we foresee its extension to a *closed loop* approach. This, of course, raises interesting research questions about the trade-off between the frequency of the update and the computational demands that will be addressed in future work.

## Supporting information

S1 Data (TXT)
